# Are inborn errors of immunity being investigated in the pediatric intensive care unit?

**DOI:** 10.1016/j.jped.2024.12.006

**Published:** 2025-02-19

**Authors:** Cecília Coelho Moraes de Brito, Patrícia Gomes de Matos Bezerra, Paula Teixeira Lyra, Maria Júlia Gonçalves de Mello

**Affiliations:** a Instituto de Medicina Integral Prof. Fernando Figueira (IMIP), Departamento de Pediatria, Recife, PE, Brazil; b Instituto de Medicina Integral Prof. Fernando Figueira (IMIP), Departamento de Imunopediatria, Recife, PE, Brazil; c Instituto de Medicina Integral Prof. Fernando Figueira (IMIP), Pós-Graduação, Recife, PE, Brazil

**Keywords:** Immune system diseases, Intensive care unit, Pediatric

## Abstract

**Objectives:**

To assess the frequency of diagnostic investigation for inborn errors of immunity (IEI) in the presence of warning signs and clinical outcomes of children hospitalized in the pediatric intensive care unit (PICU) according to age group.

**Material and methods:**

This retrospective cross-sectional study included children hospitalized in the PICU of a referral hospital over two years. Warning signs were collected according to age group (up to and over one year), and sociodemographic and clinical variables, basic investigation of IEI, follow-up by the immunology service, and hospital discharge and death were also assessed.

**Results:**

Of 680 included children, 330 (48.5%) were aged up to one year, and 350 (51.5%) were over one year. Among those aged up to one year, 108 (32.7%) had two or more warning signs, and only 22 (20.4%) were investigated for IEI. Considering the group aged over one year, 48 (13.7%) had two or more warning signs, and 21 (43.8%) were investigated for IEI. The highest number of deaths occurred among children aged up to one year with two or more warning signs (58.8%).

**Conclusions:**

Few children with warning signs were investigated for IEI, representing missed opportunities to timely diagnose IEI, which may impact the risk of death.

## Introduction

The inborn errors of immunity (IEI), also known as primary immunodeficiencies, represent a heterogeneous group of diseases with quantitative or functional changes in components of innate and adaptive immune responses.^1^ Although IEIs are considered rare,^2^ epidemiological evidence suggests that their real prevalence is underestimated.^3^ Also, advances in diagnostic methods have revealed an increasing prevalence of IEI, estimated in 1/1000 to 1/5000 live births.^2^

According to the updated classification by the International Committee of Primary Immunodeficiency Societies, over 400 IEI with 485 genetic defects were identified, encompassing a diverse group with substantial phenotypic and clinical differences between patients.^2^ Clinical manifestations may range from mild to life-threatening conditions, depending on the specific type of underlying immune defect (e.g., recurrent infections, autoimmunity, lymphoproliferation, granulomatous process, atopy, and malignancy).^4^ In this sense, a high level of diagnostic suspicion is required for investigation, emphasizing the need for a detailed clinical history and physical examination.^4^

Early identification of IEI is essential for interventions before severe infections compromise the overall health status. However, the failure to recognize these conditions remains a major challenge for clinicians worldwide, leading to high rates of underdiagnosis^5^ and emphasizing the ongoing need for medical education.^6^ A study combined data from Brazilian IEI registries with the Jeffrey Modell Foundation (JMF) and estimated that only 10% of IEI were diagnosed worldwide.^7^ The JMF first described warning signs to guide diagnosis^4^ in children and young adults in 1990,^8^ which were later adapted for Brazil by the Brazilian Immunodeficiency Group.^9^ Considering that most severe immune defects manifest in early life, 12 warning signs have been proposed for children aged under one year^10^ (Table 1).

**Table 1:** Warning signs for inborn immune errors (IEI) according to age group.^10^^,^^11^

Warning signs of inborn errors of immunity (IEI)	
In the first year of life	Ten warning signs in children
Severe or persistent fungal, viral, or bacterial infections Congenital heart defects (mainly conotruncal anomalies) Adverse reaction to live vaccine, especially Bacillus Calmette-Guérin (BCG) Delayed umbilical cord detachment (> 30 days) Autoimmune or inflammatory manifestations Persistent lymphocytopenia (< 2500 cells/mm^3^), other cytopenia, or leukocytosis without infection Family history of IEI or early death caused by infection Extensive skin lesions Persistent diarrhea Sepsis-like clinical condition without microbial isolation Hypocalcemia with or without seizures Absence of thymic shadow at X-ray	Four or more episodes of otitis within one year Two or more episodes of severe sinusitis within one year Two or more episodes of pneumonia within one year Two or more systemic infections, including sepsis Use of antibiotics for two months or more with minimal effect Difficulty in gaining weight or regular growth Recurrent skin abscesses or abscesses in internal organs Stomatitis or oral or skin candidiasis for more than two months Need for intravenous antibiotics to control infection Family history of IEI

Laboratory investigation may involve three consecutive stages: screening, advanced, and confirmatory genetic tests.^11^ Although molecular analysis is essential for a definitive diagnosis, warning signs for IEI are especially important to avoid diagnostic delays in developing countries with limited access to high-cost tests.^12^ In this sense, raising awareness of IEI and accessibility to tests should be prioritized in all health centers to change the disease course and patients’ lives. All healthcare professionals, especially those managing patients with increased risk of death (e.g., in intensive care units), should be aware of the IEI possibility, avoiding those opportunities for diagnosis are missed. Thus, this study aimed to assess the frequency of investigation for IEI in the presence of warning signs in children hospitalized in the pediatric intensive care unit (PICU).

## Material and methods

This analytical cross-sectional study was conducted in the PICU of the *Instituto de Medicina Integral Prof. Fernando Figueira* (IMIP), a quaternary referral hospital for the Brazilian Unified Health System (SUS) in northeastern Brazil. This PICU has 20 beds for clinical conditions, excluding children with trauma, cancer, or undergoing pre and postoperative cardiac surgery. The study included children aged under 15 years hospitalized in the PICU between March 1, 2020 and February 28, 2022. Children with planned admission for surgery and without complications in the post-operatory, children positive for human immunodeficiency virus (HIV), or with a pre-existing diagnosis of IEI were excluded.

Sociodemographic characteristics (i.e., age, sex, place of residence), baseline clinical variables (i.e., comorbidities, previous hospital admissions, parental consanguinity, family history of IEI or early death, and the diagnosis at hospitalization in the PICU), IEI investigation (warning signs, laboratory tests, and follow-up by the immunology service), and clinical outcomes (length of stay in the PICU, hospital discharge, and death) were collected. Medical records were analyzed, and the diagnosis at hospitalization (e.g., respiratory failure or severe respiratory distress, renal failure, sepsis, and septic shock) was defined as the main syndromic issue.

The frequency of investigation was determined for children with two or more warning signs for IEI. According to the JMF warning signs, if two or more of the signs are present, children should be investigated. The 12 signs for children up to one year include a broader spectrum of symptoms, however, does not determine the number of signs present that indicate investigation. In this study, the threshold of two signs was used as an indication of investigation, in line with what is used for the JMF warning signs.

Primary and secondary immunodeficiency investigations included basic immunological screening (i.e., blood count and serum immunoglobulin levels), whereas the secondary investigation considered HIV infection (i.e., viral load or serology). Tests were considered abnormal when immunoglobulin levels were below the third percentile for age and gestational age^13^ or in case of lymphocytopenia (total lymphocyte < 3000/μL in children aged under one year and < 1500/μL in older children)^14^ or neutropenia (total neutrophil < 1500/μL).^15^

Outcomes included missed opportunities for IEI diagnosis (i.e., when the child presented two or more warning signs but was not investigated) and frequency of deaths based on the presence or absence of signs. The frequency of abnormal screening tests for IEI and referral to the immunology service were assessed among the investigated children.

Children with potentially IEI-related genetic abnormalities dismissed diagnosis of IEI, deaths before follow-up, and without subsequent follow-up were analyzed among those who were followed up at the pediatric immunology outpatient unit.

Data were plotted into the RedCap, exported to an Excel spreadsheet, and analyzed using the Stata®13.0 software. The Pearson's chi-squared test compared variables and outcomes, and statistical significance was set at *p* < 0.05 for all tests.

This study followed the Resolution 466/2012 of the Brazilian National Health Council and the Declaration of Helsinki. Risks were minimized by ensuring the anonymity of sensitive data in medical records according to the General Data Protection Law. This study was approved by the ethics committee of IMIP, n° 59266422.8.0000.5201.

## Results

Of the 860 children hospitalized in the PICU during the study, 142 did not meet the eligibility criteria, and 38 (5.3%) were considered losses due to the unavailable medical record. Thus, 680 children were included; 330 (48.5%) were aged up to one year and 350 (51.5%) over 1 year. Sociodemographic and clinical characteristics according to age groups are shown in Table 2.

**Table 2:** Sociodemographic and clinical characteristics according to the age group of children hospitalized in the pediatric intensive care unit (PICU) of the Instituto de Medicina Integral Professor Fernando Figueira (IMIP) from March 1, 2020, to February 28, 2022.

Variables	Aged up to 1year (*n* = 330)	Aged over 1 year (*n* = 350)
Age (months)		
Range	0 – 11.7	12.4 – 178.0
Mean ± standard deviation	3.2 ± 3.1	73.2 ± 48.4
Median	2.1	64.5
Sex, n (%)		
Male	199 (60.3)	167 (47.7)
Female	131 (39.7)	183 (52.3)
Place of residence		
Recife and metropolitan region	137 (41.5)	189 (54.0)
Inland and other states	193 (58.5)	161 (46.0)
Comorbidities, n (%)		
Congenital heart disease	76 (23.0)	30 (8.6)
Genetic syndrome	42 (12.7)	19 (5.4)
Chronic kidney disease	11 (3.3)	34 (9.7)
History of prematurity	51 (15.4)	6 (1.7)
Asthma or recurrent wheezing	2 (0.6)	60 (17.1)
Chronic non-progressive encephalopathy	26 (7.9)	49 (14.0)
Autoimmune diseases	2 (0.6)	13 (3.7)
Endocrinopathy	5 (1.5)	32 (9.1)
Other*	26 (7.9)	34 (9.7)
Diagnosis at hospitalization in the PICU, n (%)		
Respiratory failure or severe respiratory distress	139 (42.1)	153 (43.7)
Sepsis or septic shock	119 (36.1)	108 (30.8)
Decompensation of congenital heart disease	23 (7.0)	16 (4.6)
Diseases of the neonatal period	32 (9.7)	0 (0)
Renal failure	2 (0.6)	27 (7.7)
Diabetic ketoacidosis	0 (0)	23 (6.6)
Other**	15 (4.5)	23 (6.6)

The total sum of comorbidities is higher than the number of children because some had more than one comorbidity.

*Other comorbidities in children aged up to one year included primary malnutrition (seven), pneumopathy (six), inborn errors of metabolism (five), laryngomalacia (three), cleft palate (two), and osteopetrosis (one).

*Other comorbidities in children aged over one year included neoplasia (ten), pneumopathy (eight), sickle cell anemia (five), primary malnutrition (three), inborn errors of metabolism (three), hepatopathy (two), epidermolysis bullosa (one), and autism spectrum disorder (one).

⁎⁎ Other diagnoses included hypovolemic shock, acute neurological deficit, and status epilepticus.

The main cause of hospitalization was severe respiratory failure or distress, followed by sepsis or septic shock in children aged up to (42.1% and 36.1%, respectively) and over one year (43.7% and 30.8%, respectively). Among children aged up to one year, 222 (67.3%) had less than two warning signs for IEI, 108 (32.7%) had two or more signs, and 102 (30.9%) deaths were registered. Also, 302 (86.3%) children aged over one year had less than two warning signs, 48 (13.7%) had two or more signs, and 70 (20%) deaths were registered (Table 3).

**Table 3:** History, warning signs of inborn errors of immunity (IEI), length of stay, and discharge data according to the age group of children hospitalized in the pediatric intensive care unit (PICU) of the Instituto de Medicina Integral Professor Fernando Figueira (IMIP) from March 1, 2020, to February 28, 2022.

Variables	Aged up to 1 year (*n* = 330)		Aged over 1 year (*n* = 350)	
Consanguinity, n (%)				
Yes	2	(0.6)	3	(0.9)
No	14	(4.2)	13	(3.7)
No record	314	(95.2)	334	(95.4)
Family history of IEI or early death in the family, n (%)				
Yes	1	(0.3)	3	(0.9)
No	6	(1.8)	6	(1.7)
No record	323	(97.9)	341	(97.4)
Previous hospital admissions, n (%)				
Inward only	42	(12.7)	108	(30.9)
In PICU	48	(14.6)	52	(14.9)
No hospitalization	183	(55.5)	125	(35.7)
No record	57	(17.3)	65	(18.6)
Comorbidities, n (%)				
No comorbidities	153	(46.4)	106	(30.3)
One or more	177	(53.6)	244	(69.7)
Warning signs of IEI, n (%)				
Less than two warning signs	222	(67.3)	302	(86.3)
Two or more warning signs	108	(32.7)	48	(13.7)
Length of PICU stay (days)				
Range	1 – 262		1 – 89	
Mean ± SD	15.8 ± 24.5		10.4 ± 13.3	
Median	8		5	
Discharge, n (%)				
Hospital discharge	228	(69.1)	280	(80.0)
Death	102	(30.9)	70	(20.0)

PICU, pediatric intensive care unit; IEI, inborn errors of immunity; n, absolute frequency; SD, standard deviation.

Table 4 shows the results of diagnostic investigations, follow-up by the immunology service, and outcomes (i.e., discharge or death) based on age groups and the presence of two or more warning signs. Children aged over one year with two or more warning signs were more investigated than children up to one year (*p* = 0.003).

**Table 4:** Type of investigation and discharge data according to age group and presence of warning signs of inborn errors of immunity (IEI) in children hospitalized in the pediatric intensive care unit (PICU) of the Instituto de Medicina Integral Professor Fernando Figueira (IMIP) from March 1, 2020, to February 28, 2022.

Variables	Warning signs for IEI			
	Aged up to 1 year (*n* = 330)		Aged over 1 year (*n* = 350)	
	Less than two signs	Two or more signs	Less than two signs	Two or more signs
Children, n (%)	222 (67.3)	108 (32.7)	302 (86.3)	48 (13.7)
Investigation, n (%)				
Not performed		74 (68.5)		22 (45.8)
HIV serology		12 (11.1)		5 (10.4)
Initial IEI screening		22 (20.4)		21 (43.8)
Test results, n (%)				
Within normal range		18 (81.8)		16 (76.2)
Abnormal		4 (18.2)		5 (23.8)
Follow-up by the immunology service, n (%)				
No follow-up		7 (31.8)		4 (19.0)
Follow-up without a confirmed diagnosis		4 (18.2)		9 (42.8)
Dismissed IEI		0 (0)		1 (4.9)
Genetic abnormalities^a^		2 (9.1)		2 (9.5)
Death before follow-up		9 (40.1)		5 (23.8)
Discharge, n (%)				
Hospital discharge	180 (81.1)	48 (44.4)	248 (82.1)	32 (66.7)
Death	42 (18.9)	60 (58.8)	54 (17.9)	16 (33.3)

n, absolute frequency; IEI, inborn errors of immunity; HIV, human immunodeficiency virus.

a Of children who presented abnormalities in genetic tests, two were diagnosed with osteopetrosis, and two presented variants of uncertain significance that might be associated with IEI.

From both age groups, 22.9% of children had two or more warning signs for IEI (indicating the need for investigation), and 27.6% underwent the minimum tests for initial investigation, representing missed opportunities for the diagnosis. From both groups, among those who performed the initial investigation, 20.9% of them presented abnormal initial results. One child over one year who initially had abnormal results was followed up and subsequently presented results within the normal range.

Children with and without two or more warning signs for IEI presented statistically significant differences in the frequency of deaths in both age groups (*p* < 0.01 for children up to one year and *p* = 0.013 for children over 1 year) (Table 4).

## Discussion

This study showed that only a minority of children with two or more warning signs for IEI were investigated in a maternal and child referral center in northeastern Brazil.

Although useful and widely disseminated, the warning signs for IEI have not been prospectively validated.^16^ A study assessing the presence of 10 warning signs in children with IEI showed low specificity (23%) and relatively low sensitivity (63%)^17^ for detecting IEI, with positive family history being the strongest identifier (18 folds more prevalent in children with confirmed diagnosis).^18^ In the present study, only 2.4% of medical records presented a family history of IEI or early death, emphasizing the need for a detailed anamnesis to increase the diagnosis. Of those, 4 patients had a positive history of IEI or early death in the family. One patient was up to one year old and was not investigated for IEI. The other three patients had more than one year, one of them died and the other two were investigated and followed up in immunology but did not have a confirmed diagnosis.

In the PICU routine investigation for IEI is not frequently done, and the data on the prevalence of this condition is the PICU unit is not well known but is expected to be higher than in the general population.^19^ In this study about 14% of the patients had been admitted in ICU previously, but there was no specific investigation protocol for this group. However, it is possible that the patients in this service had underlying conditions (such as the history of prematurity and cardiac congenital diseases), that could justify the previous admissions.

Severe infections are the first sign of IEI in over 75% of patients^20^ and an important cause of hospitalizations in intensive care units. In this study, sepsis and septic shock were the second leading cause of hospitalization for all age groups. The association of sepsis with IEI has been analyzed, revealing pathogenic variants in genes associated with IEI in 20% of children with sepsis who were previously healthy.^21^ Also, genetic variants associated with immunodeficiency were identified in up to 61% of children with severe sepsis.^22^

Although other protocols including recently identified IEI changes were proposed for its diagnosis,^23^ they are generally complex and difficult to apply. The ten warning signs are an important tool to raise awareness of IEI and reduce the time between symptom onset and diagnosis,^17^ especially in countries with limited access to complex tests, such as Brazil. Despite being conducted in a quaternary teaching hospital with one of the three pediatric immunology services of the state of Pernambuco, this study showed that children were not regularly investigated. This finding reinforced data from another study conducted in a PICU where children were often not investigated despite indications.^24^

The number of deaths was statistically different between children with and without warning signs for IEI in both age groups, possibly due to the severity criteria of the warning signs (e.g., severe infections). However, some children could have an underlying disease justifying the increased number of deaths, considering that children with IEI are more frequently hospitalized with longer hospital stays and higher mortality rates than the general population.^25^

The highest number of deaths was found in children aged up to one year with two or more warning signs. Similarly, a study in the Russian population reported a mortality rate of 42% in children aged under two years with IEI.^26^ Nine (40.1%) children aged up to one year with two or more warning signs who underwent initial investigation for IEI died before progressing to further investigation, possibly because most severe IEI manifests early, and some are pediatric emergencies.^10^ Children aged up to one year with two or more warning signs were less frequently investigated than those over one year, corroborating literature on IEI investigation among children aged up to one year and reinforcing the need for specific signs for this age group.^10^^,^^20^^,^^27^

Laboratory tests used in the study (i.e., blood count and serum immunoglobulin levels) represent screening tools for IEI and do not dismiss its possibility when results are within normal range. However, 20,9% of the children with two or more signs investigated showed abnormalities in initial tests, including neutropenia or lymphocytopenia and reduced immunoglobulin levels. If this data was extrapolated to all children with two or more warning signs, 32 of the 156 with two or more warning signs could have abnormalities, representing missed opportunities in investigating and diagnosing children with IEI. Although these laboratory abnormalities do not confirm IEI diagnosis, children should be followed up and have the tests repeated after the current infection.

Although not widely available, genetic sequencing tests were performed in this PICU for some children with two or more warning signs, with two (9.1%) aged up to 1 year and two (9.5%) over 1-year showing abnormalities. Of these, two were diagnosed with osteopetrosis (one aged over and one up to 1 year), which is described to be associated with IEI.^2^ The other two showed variants of uncertain significance that might be associated with IEI.

Considering the retrospective design using medical records, this study had some limitations. Most PICU records did not provide a family history of IEI or early death in the family, potentially underestimating the number of patients with one or more warning signs. Also, this study was conducted during the COVID-19 pandemic, which could have changed the hospitalization pattern, especially during lockdown, when children were less exposed to infectious agents due to social isolation.^28^ The unavailability of specific laboratory tests (e.g., dihydrorhodamine assay and genetic sequencing) hindered the diagnosis of IEI. Thus, further studies are needed to accurately assess the frequency of IEI among children hospitalized in the PICU.

Considering that missed opportunities for diagnosing IEI were observed, healthcare professionals should be aware of this possibility (especially in patients hospitalized in intensive care units) to enhance the early diagnosis, improving the quality of life and reducing morbidity and mortality among children with IEI.

## Conflicts of interest

The authors declare no conflicts of interest.
